# Multitarget Test for Emerging Lyme Disease and Anaplasmosis in a Serosurvey of Dogs, Maine, USA

**DOI:** 10.3201/eid1705.100408

**Published:** 2011-05

**Authors:** Peter W. Rand, Eleanor H. Lacombe, Susan P. Elias, Bruce K. Cahill, Charles B. Lubelczyk, Robert P. Smith

**Affiliations:** Author affiliation: Maine Medical Center Research Institute, South Portland, Maine, USA

**Keywords:** vector-borne infections, Lyme disease, anaplasmosis, tick-borne infections, bacteria, canine serosurvey, Anaplasma phagocytophilum, Borrelia burgdorferi, SNAP 4Dx test, dispatch

## Abstract

To determine if the range of deer ticks in Maine had expanded, we conducted a multitarget serosurvey of domestic dogs (*Canis lupus familiaris)* in 2007. An extension of exposure to *Borrelia burgdorferi* to the northern border and local transmission of *Anaplasma phagocytophilum* throughout southern areas was found.

Over the past 2 decades, the range of *Ixodes scapularis*, the deer tick, vector of Lyme disease, anaplasmosis, babesiosis, and deer tick virus infections, has expanded in northern New England. Because Lyme disease and anaplasmosis affect humans and dogs (*Canis lupus familiaris*), serosurveys of canids have proved useful for monitoring emergence of these infections. Sample selection may be confounded when dogs that are remotely exposed, vaccinated, or treated with topical acaricides are included. In recent years, however, the advent of a multitarget, in-clinic test kit (SNAP 4Dx; IDEXX Laboratories, Westbrook, ME, USA) has increased the scope and efficiency of these serosurveys. The SNAP 4Dx tests for heartworm antigen and antibodies to *Borrelia burgdorferi*, *Anaplasma phagocytophilum*, and *Ehrlichia canis* on 3 drops of blood. Its sensitivity and specificity for antibodies against *B. burgdorferi* and *A. phagocytophilum* exceed 98% (*1**,**2*).

In Maine, deer ticks were first reported at a coastal site in 1988 and have since spread inland (*3*). Lyme disease has become a major public health problem; reported human cases reached 169 per 100,000 population in 1 mid-coastal county in 2008. Human cases of anaplasmosis and babesiosis are also being reported (*4*). In 1990, we conducted a statewide serosurvey to map *B. burgdorferi*–positive dogs and to correlate their distribution with reported human cases. Four percent of 828 samples were seropositive for *B. burgdorferi*, 89% of which were from dogs residing within 20 miles of the coast. No positivity was found among 102 dogs in the northern half of the state (*5*). Given the widespread acceptance of SNAP 4Dx tests by Maine veterinarians, we resurveyed dogs statewide in 2007 for exposure to *B. burgdorferi* and *A. phagocytophilum.* Data from questionnaires to veterinarians and dog owners enabled assessment of the influence of the use of Lyme vaccines and topical acaricides on canine serologic test results.

## The Study

From 87 veterinary clinics solicited in 2007, we selected 47 on the basis of their size and geographic distribution. Each was supplied with 15–30 SNAP 4Dx kits (contributed by IDEXX Laboratories). Veterinarians were instructed to obtain samples from all dogs routinely tested for heartworm. In northern areas, where heartworm is rarely tested for, they were asked to collect samples from dogs undergoing surgery. They recorded each dog’s age, town of residence, Lyme disease vaccination status (ever or never vaccinated), and the test results. Each dog owner completed a form (99.6% response rate) to describe the dog, its function, history of unexplained lameness, travel history (town, state, visited within the past year), history of tick infestation, and use of tick control products (yes or no).

We summarized test results to town and county levels. We used Spearman rank correlation tests to examine associations between canine seropositivity, human Lyme disease cases reported to the Maine Center for Disease Control and Prevention (Augusta, ME, USA) (*4*), and the number of deer ticks submitted to our laboratory in 2007. We used *B. burgdorferi* and *A. phagocytophilum* test results and questionnaire responses to cross-tabulate responses and calculate the likelihood (odds ratios) of *B. burgdorferi* and *A. phagocytophilum* positivity as a function of risk factors by using χ^2^ tests of association. We considered differences significant at p<0.05. Analyses were conducted by using SAS version 9.2 for Windows (SAS, Cary, NC, USA).

**Figure 1:**
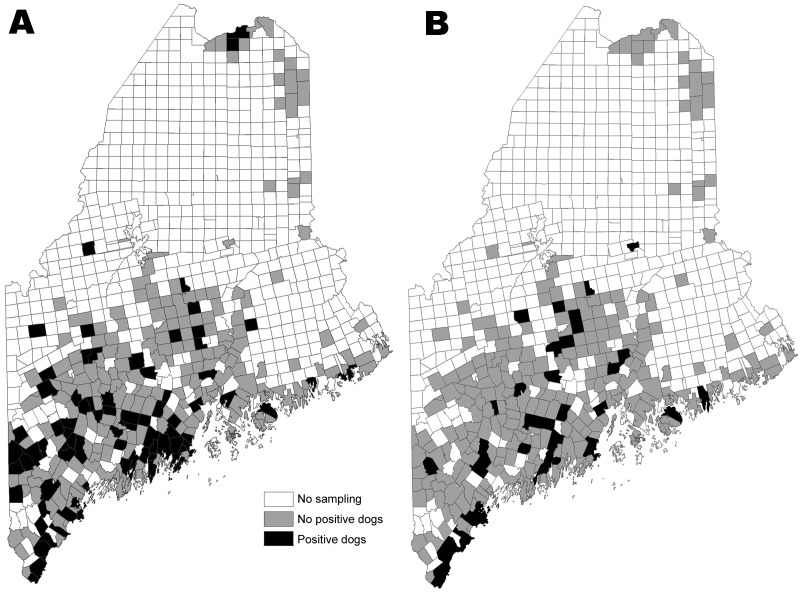
Towns where dogs were tested for seropositivity to *Borrelia burgdorferi* (A) and *Anaplasma phagocytophilum* (B) in a statewide serosurvey of domestic dogs, Maine, USA, 2007.

**Figure 2:**
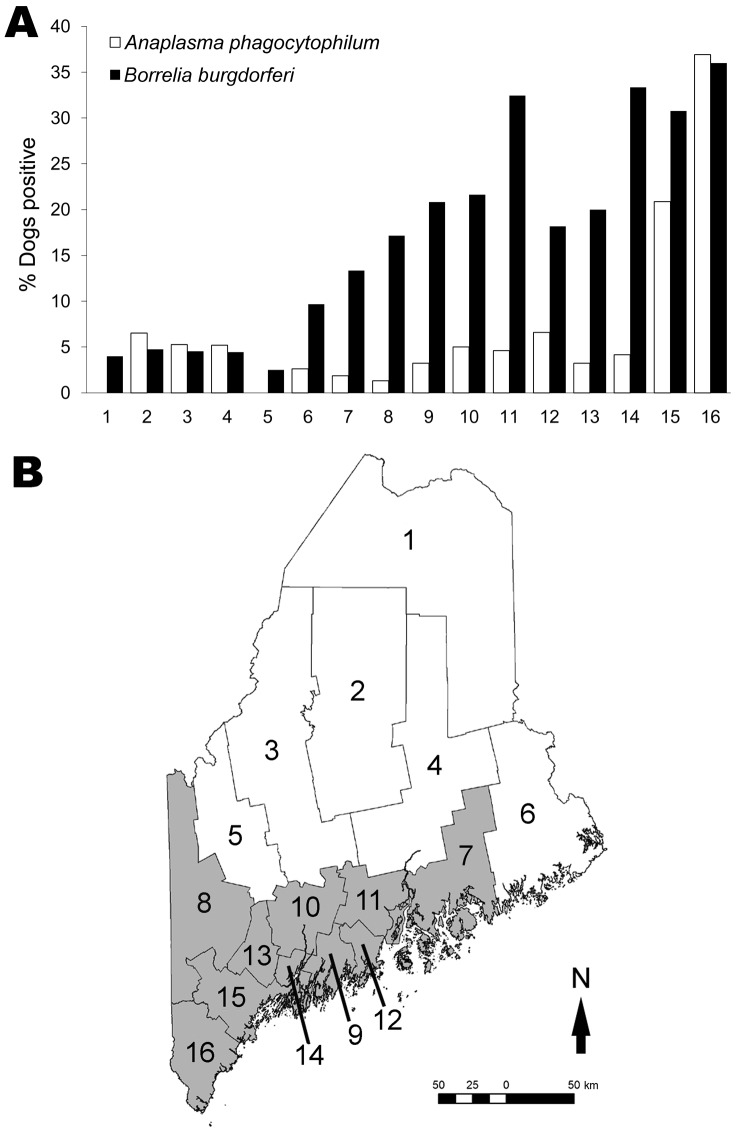
A) Canine seroprevalence for *Anaplasma phagocytophilum* and, in dogs never vaccinated against Lyme disease, for *Borrelia burgdorferi* in Maine counties arranged north to south, 2007. B) Maine counties, with the 10 tick-abundant counties used in the analyses shaded in gray. Counties: 1, Aroostook; 2, Piscataquis; 3, Somerset; 4, Penobscot; 5, Franklin; 6, Washington; 7, Hancock; 8, Oxford; 9, Waldo; 10, Kennebec; 11, Knox; 12, Lincoln; 13, Androscoggin; 14, Sagadahoc; 15, Cumberland; 16, York.

Of 1,087 dogs tested across Maine’s 16 counties, 12.7% were *B. burgdorferi*–positive and 7.1% were *A. phagocytophilum*–positive (Table 1); 1.9% were co-infected. The distribution of all dogs seropositive for either pathogen is shown by town in Figure 1. At the county level, canine *B. burgdorferi* seropositivity among unvaccinated dogs correlated positively with the number of human Lyme disease cases reported for 2007(ρ_Spearman_ = 0.84; p<0.0001) and the number of deer ticks submitted to our laboratory for identification (ρ_Spearman_ = 0.63; p = 0.009). In Figure 2, which shows statewide distributions by county north to south, only unvaccinated dogs are included in *B. burgdorferi*–positive data shown. Dogs had been exposed to *A. phagocytophilum* in all but 2 northern counties. At the town level, remarkably higher levels of canine *A. phagocytophilum* seropositivity were found in southern coastal Cape Elizabeth (Cumberland County) (76.5%, n = 17) and York (York County) (58.0%, n = 19) than in towns in their immediate vicinity.

**Table 1:** Canine seroprevalence of *Borrelia burgdorferi* and *Anaplasma phagocytophilum* and Lyme diseases vaccination status, Maine, USA, 2007

County	No. dogs tested	B. burgdorferi*			A. phagocytophilum*			Lyme disease vaccination status	
		No. negative	No. (%) positive		No. negative	No. (%) positive		No. reporting	No. (%) vaccinated
Aroostook	59	56	3 (5.1)		59	0		59	9 (15.3)
Piscataquis	46	44	2 (4.3)		43	3 (6.5)		44	23 (52.3)
Somerset	57	55	2 (3.5)		54	3 (5.3)		55	33 (60.0)
Penobscot	77	73	4 (5.2)		73	4 (5.2)		75	30 (40.0)
Franklin	78	73	5 (6.4)		78	0		78	38 (48.7)
Washington	38	35	3 (7.9)		37	1 (2.6)		37	6 (16.2)
Hancock	54	46	8 (14.8)		53	1 (1.9)		54	24 (44.4)
Oxford	76	66	10 (13.2)		75	1 (1.3)		76	41 (53.9)
Waldo	62	57	5 (8.1)		60	2 (3.2)		62	38 (61.3)
Kennebec	120	106	14 (11.7)		114	6 (5.0)		119	82 (68.9)
Knox	87	67	20 (23.0)		83	4 (4.6)		81	44 (54.3)
Lincoln	91	75	16 (17.6)		85	6 (6.6)		85	63 (74.1)
Androscoggin	62	53	9 (14.5)		60	2 (3.2)		62	27 (43.5)
Sagadahoc	24	22	2 (8.3)		23	1 (4.2)		24	21 (87.5)
Cumberland	91	78	13 (14.3)		72	19 (20.9)		88	62 (70.5)
York	65	42	22 (34.4)		41	24 (36.9)		59	34 (57.6)
Total	1,087	948	138 (12.7)		1,010	77 (7.1)		1,058	575 (54.3)

*Tested by using SNAP 4Dx test kit (IDEXX Laboratories, Westbrook, ME, USA).

Overall, 54.3% of the dogs had been vaccinated against Lyme disease. Never-vaccinated dogs were 1.5× as likely to be seropositive for *B. burgdorferi* than were vaccinated dogs (15.3% vs. 9.9%; p = 0.008) (Table 2). Vaccine use was higher in 10 southern counties where Lyme disease has become endemic (Figure 2, panel B) than in the 6 northern counties where it is emerging (63.9% vs. 42.7%; p<0.0001) and correlated positively with the number of deer ticks submitted to our laboratory for identification in 2007 (n = 16 counties, ρ_Spearman_ = 0.63; p = 0.009). Two thirds of respondents said that their dogs had traveled out of town; however, no associations were found between *B. burgdorferi* or *A. phagocytophilum* seropositivity and the dog’s travel history. Three of 59 dogs in the northernmost county of Maine were *B. burgdorferi*–positive, 1 of which had never traveled beyond its home town. Eighty-three percent of owners reported using acaricides. Despite the effective protection reported for topical acaricides (*6**,**7*), no difference in seropositivity between treated and untreated dogs was evident on the basis of their reported use (Table 2). Unexplained lameness was 1.5× more likely in dogs that were only *A. phagocytophilum*–positive than in those only *B. burgdorferi*–positive (40.0% vs. 26.5%; p*<*0.06).

**Table 2:** Risk factors vs. canine seroprevalence of *Anaplasma phagocytophilum* and *Borrelia burgdorferi*, Maine, USA, 2007*

Variable	No. dogs	*Borrelia burgdorferi*				*Anaplasma phagocytophilum*		
No. (%) positive	OR (95% CI)	p value†	No. (%) positive	OR (95% CI)	p value†
Lyme vaccine								
Yes	575	57 (9.9)				49 (8.5)		
No	483	75 (15.3)	1.5 (1.1–2.1)	0.008		23 (4.8)	0.6 (0.3–0.9)	0.02
Travel‡								
All dogs								
None	357	49 (13.7)				27 (7.6)		
>1	730	89 (12.2)	0.9 (0.6–1.3)	NS		50 (6.9)	0.9 (0.6–1.5)	NS
Unvaccinated dogs								
None	163	21 (12.9)				8 (4.9)		
>1	320	53 (16.6)	1.3 (0.8–2.3)	NS		15 (4.7)	0.9 (0.4–2.3)	NS
Tick control products								
All dogs								
No	182	20 (11.0)				7 (3.9)		
Yes	899	115 (12.9)	1.2 (0.7–2.0)	NS		66 (7.4)	2.0 (0.9–4.4)	0.08
Unvaccinated dogs								
No	124	13 (10.5)				3 (2.4)		
Yes	350	59 (16.9)	1.7 (0.9–3.3)	0.09		18 (5.1)	2.2 (0.6–7.6)	NS
History of unexplained lameness								
No	877	97 (11.1)				42 (4.8)		
Yes	191	40 (20.9)	2.1 (1.4–3.2)	0.0002		28 (15.2)	3.6 (2.1–5.9)	<0.0001

*A total of 1,087 dogs were tested. OR, odds ratio; CI, confidence interval; NS, not significant. †Significance based on Pearson χ^2^ statistic with 1 degree of freedom. ‡Trips away from town of residence.

## Conclusions

This study demonstrates that risk of contracting Lyme disease has reached northernmost Maine and that anaplasmosis is now being transmitted to dogs throughout the lower half of the state. The study expands on nationwide SNAP 4Dx data documenting *B. burgdorferi* and *A. phagocytophilum* positivity in the southern half of the state (*8*). In southern coastal Maine, overabundant white-tail deer, appropriate habitat, and maritime climate all contribute to high densities of *I. scapularis* ticks (*3*) and consequent disease transmission; thus, the remarkably high level of *A. phagocytophilum* seroreactivity observed in the southern coastal towns of Cape Elizabeth and York calls for further work to understand the dynamics of the intense local emergence of this pathogen. The higher level of unexplained lameness in *A. phagocytophilum*–positive dogs than in *B. burgdorferi*–positive dogs is consistent with findings by Beall et al. (*9*), who reported a 2.6× greater incidence of *A. phagocytophilum* seroreactivity than *B. burgdorferi* seroreactivity among 32 lame, non–co-infected dogs in Minnesota who were suspected of having either disease. The lameness also reflects the high percentage of *B. burgdorferi* positivity among asymptomatic dogs (*10*). That *B. burgdorferi* and *A. phagocytophilum* seropositivity rates were essentially identical between dogs who had a history of travel and those who did not lessens concern about travel as a confounding variable, an exposure difficult to interpret in any event, given the spotty distribution of ticks even where Lyme disease is endemic (*11*).

In a recent study, Hamer et al. (*12*) reported that a serosurvey of canines for *B. burgdorferi* is ineffective in a region that includes areas with little *B. burgdorferi* transmission, and less informative than analysis of ticks removed from dogs. The authors referred to the confounding influence of tick chemoprophylaxis. Our inability to detect an effect of topical acaricides may reflect their ubiquitous use for flea control and a lack of information on the frequency of their use. Although the widespread use of protective measures now complicates interpretation of serosurveys of canines, in selected dogs the availability of a reliable, multitarget test that is used routinely nationwide (*8*) remains a valuable and cost-effective method for documenting transmission of the agents of Lyme borreliosis and anaplasmosis, particularly in areas where disease is emerging.

## References

[1] O’Connor TP, Esty KJ, Hanscom JL, Shields P, Phillip MD. Dogs vaccinated with common Lyme disease vaccines do not respond to IR6, the conserved immunodominant region of the VlsE surface protein of *Borrelia burgdorferi.* Clin Diagn Lab Immunol. 2004;11:458–62. 15138170 10.1128/CDLI.11.3.458-462.2004 PMC404571

[2] Chandrashekar R, Mainville CA, Beall MJ, O’Connor T, Eberts M, Alleman AR, Performance of a commercially available in-clinic ELISA for the detection of antibodies against *Anaplasma phagocytophilum, Ehrlichia canis*, and *Borrelia burgdorferi* and *Dirofilaria immitis* antigen in dogs. Am J Vet Res. 2010;71:1443–50. 10.2460/ajvr.71.12.1443 21117995

[3] Rand PW, Lacombe EH, Dearborn R, Cahill B, Elias S, Lubelczyk CB, Passive surveillance in Maine, an area emergent for tick-borne diseases. J Med Entomol. 2007;44:1118–29. 10.1603/0022-2585(2007)44[1118:PSIMAA]2.0.CO;2 18047214

[4] Robbins A. Reportable infectious diseases in Maine: 2008 summary. Augusta (ME): Maine Center for Disease Control and Prevention; 2009.

[5] Rand PW, Smith RP Jr, Lacombe EH. Canine seroprevalance and the distribution of *Ixodes dammini* in an area of emerging Lyme disease. Am J Public Health. 1991;81:1331–4. 10.2105/AJPH.81.10.1331 1928538 PMC1405313

[6] Blagburn BL, Spencer JA, Billeter SA, Drazenovich NL, Butler JM, Land TM, Use of imidacloprid-permethrin to prevent transmission of *Anaplasma phagocytophyilum* from naturally-infected *Ixodes scapularis* ticks to dogs. Vet Ther. 2004;5:212–7. 15578453

[7] Blagburn BL, Spencer JA, Butler JM, Land TM, Billeter SA, Dykstra CC, Prevention of transmission of *Borrelia burgdorferi* and *Anaplasma phagocytophilum* from ticks to dogs using K9 Advantix and Frontline Plus applied 25 days before exposure to infected ticks. Intern J Appl Res Vet Med. 2005;3:69–75.

[8] Bowman D, Little SE, Lorentzen L, Shields J, Sullivan M, Carlin EP. Prevalence and geographic distribution of *Dirofilaria immitis, Borrelia burgdorferi, Ehrlichia canis,* and *Anaplasma phagocytophilum* in dogs in the United States: results of a national clinic-based serologic survey. Vet Parasitol. 2009;160:138–48. 10.1016/j.vetpar.2008.10.093 19150176

[9] Beall MJ, Chandrashekar R, Eberts MD, Cyr KE, Diniz PP, Mainville C, Serological and molecular prevalence of *Borrelia burgdorferi*, and *Ehrlichia* species in dogs from Minnesota. Vector Borne Zoonotic Dis. 2008;8:455–64. 10.1089/vbz.2007.0236 18302532

[10] Levy SA, Magnarelli LA. Relationship between development of antibodies to *Borrelia burgdorferi* in dogs and the subsequent development of limb/joint borreliosis. J Am Vet Med Assoc. 1992;200:344–7. 1548169

[11] Pardanani N, Mather TN. Lack of spatial autocorrelation in fine-scale distributions of *Ixodes scapularis* (Acari: Ixodidae). J Med Entomol. 2004;41:861–4. 10.1603/0022-2585-41.5.861 15535613

[12] Hamer SA, Tsao JI, Walker ED, Mansfield LS, Foster ES, Hickling GJ. Use of tick surveys and serosurveys to evaluate pet dogs as a sentinel species for emerging Lyme disease. Am J Vet Res. 2009;70:49–56. 10.2460/ajvr.70.1.49 19119948

